# Supplementary material for: The N-terminus of Sec61p plays key roles in ER protein import and ERAD
Source: PLoS One. 2019 Apr 24;14(4):e0215950. doi: 10.1371/journal.pone.0215950 (PMC6481919; doi:10.1371/journal.pone.0215950)
Supplement: S1 Fig — (PDF) [file pone.0215950.s001.pdf]

a. Sec61p (Ac-SS)

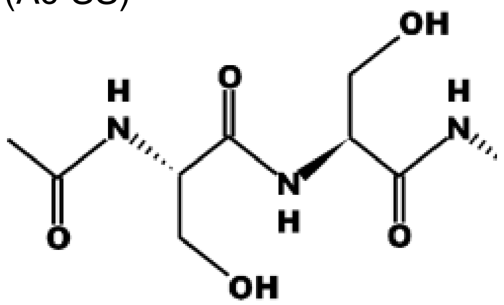

b. Sec61S2Yp (Ac-MYS)

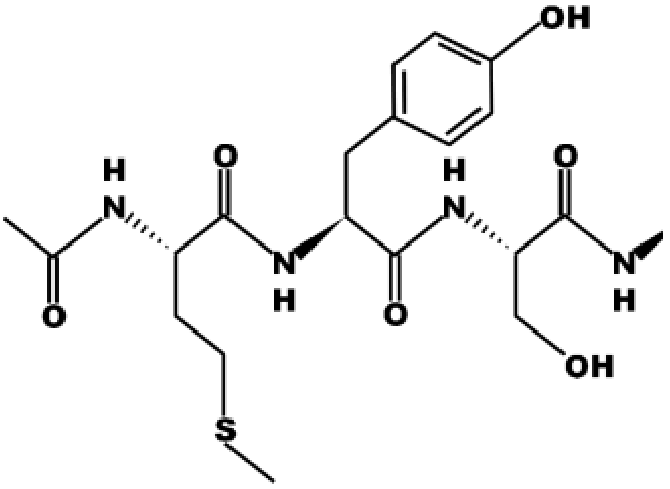

S1 Fig. N-acetylated N-termini of Sec61 and Sec61S2Y. a. Acetylated N-terminal of wild type Sec61p in *S. cerevisiae*. **b.** Acetylated N-terminal of mutant S2Y Sec61p in which Ser2 is replaced by Tyr. The initiator Met is not cleaved and is acetylated, forming a bulky N-terminus.
